# Platelet Function and Therapeutic Applications in Dogs: Current Status and Future Prospects

**DOI:** 10.3390/ani10020201

**Published:** 2020-01-25

**Authors:** Laura Cortese, Pete W. Christopherson, Alessandra Pelagalli

**Affiliations:** 1 Department of Veterinary Medicine and Animal Productions, Division of Internal Medicine, University of Naples Federico II, Via Delpino, 1, 80137 Naples, Italy; lcortese@unina.it; 2 Department of Pathobiology, Auburn University College of Veterinary Medicine, Auburn University, Auburn, AL 36849, USA; chrispw@auburn.edu; 3 Department of Advanced Biomedical Sciences, University of Naples “Federico II”, 80131 Naples, Italy; 4 Institute of Biostructures and Bioimaging (IBB), National Research Council (CNR), 80131 Naples, Italy

**Keywords:** dog, platelets, physiology, therapy, regenerative medicine

## Abstract

**Simple Summary:**

Platelets, small blood cells produced from megakaryocytes mainly in the bone marrow, are implicated not only in hemostasis, but also in different physiological and pathophysiological processes. This paper reviews canine platelet structure and functions, including an accurate description of the numerous surface receptors involved in the mechanisms of platelet adhesion and aggregation. In addition, we describe the most important canine platelet disorders that can affect platelet numbers (thrombocytopenias and thrombocytosis) or platelet function (thrombopathias). Platelet-related tests are vital in the analysis of primary hemostatic disorders and for this reason, we also discuss the efficacy of various tests in the diagnosis of canine platelet disorders. Finally, innovative therapeutic approaches based on the use of platelets and their derivatives for the treatment of various canine diseases such as inflammatory conditions (tendinitis and osteoarthritis) and for wound healing and tissue repair are reported.

**Abstract:**

Significant progress has been made in the functional characterization of canine platelets in the last two decades. The role of canine platelets in hemostasis includes their adhesion to the subendothelium, activation, and aggregation, leading to primary clot formation at the site of injury. Studies on canine platelet function and advancements in laboratory testing have improved the diagnosis and understanding of platelet-related disorders as well as the knowledge of the mechanisms behind these diseases. This review focuses on the most recent discoveries in canine platelet structure, function, and disorders; and discusses the efficacy of various tests in the diagnosis of platelet-related disorders. With the relatively recent discovery of angiogenetic and reparative effects of growth factors found in platelets, this review also summarizes the use of canine platelet-rich plasma (PRP) alone or in association with stem cells in regenerative therapy. The characterization of proteomic and lipidomic profiles and development of platelet gene therapy in veterinary species are areas of future study with potential for major therapeutic benefits.

## 1. Introduction

Platelets are tiny yet complex cytoplasmic fragments that play a crucial role in hemostasis. Recent studies have found additional platelet activities, including roles in both physiologic (tissue repair, wound remodeling, and antimicrobial host defense) [1,2,3] and pathologic conditions (thrombosis, atherosclerosis, chronic inflammatory diseases, and cancer) [4,5,6,7]. Characterization of both platelet morphology and function have shed light on many aspects of their role in clinical diseases and of their response to pharmacological therapy.

Previous studies have demonstrated differences in platelet morphology, internal organelle organization, and activity among different animal species. Functional platelet abnormalities can occur with normal platelet counts and include conditions with altered platelet adhesion, spreading, granule secretion, and/or aggregation [8].

Canine platelet function studies enhance knowledge of normal and altered hemostasis in dogs and can offer insights into human disorders, which can aid in the development of new therapeutic approaches to certain diseases. For example, a prominent therapeutic role has recently been attributed to platelet proteins, including well-characterized platelet-derived growth factor (PDGF), transforming growth factor (TGF), platelet factor interleukin (IL), platelet-derived angiogenesis factor (PDAF), vascular endothelial growth factor (VEGF), epidermal growth factor (EGF), insulin-like growth factor (IGF), and fibronectin contained in α-granules and in other internal platelet structures. The high content of growth factors (GFs) in platelets plays an important role in coagulation, immune responses, angiogenesis, and the healing of damaged tissues. The high therapeutic value of such growth factors (GFs), in association with stem cells in treating various diseases has been documented [9].

Despite several limitations (limited use of animals, inconstant number of GFs, etc.), the recent development of platelet treatment protocols in veterinary regenerative medicine has demonstrated the role of platelet-rich plasma (PRP) in activating dormant stem cells and thus making treatment more efficacious [10,11].

This paper reviews canine platelet function, various platelet receptors, and potential therapeutic impact of platelets/platelet derived products on various diseases.

## 2. Structure and Function of Platelets in Dogs

**Figure 1:**
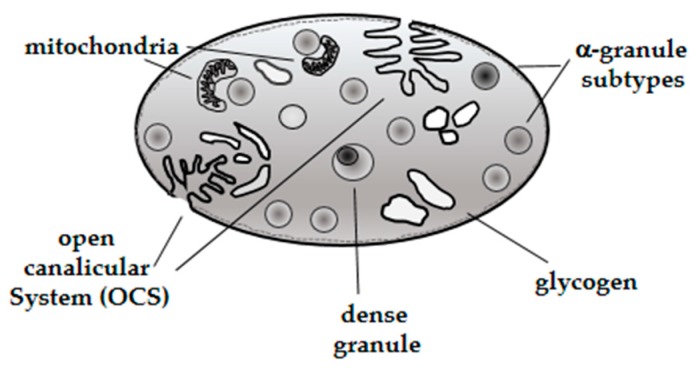
Schematic diagram of a dog platelet.

Platelets are tiny anucleate cell fragments circulating in the blood with a relatively complex structure and morphology. Canine platelet structure and anatomy has been characterized by light and electron microscopy techniques; the latter revealed a more detailed internal structure. For instance, light microscopy showed platelet size and shape and the presence of cytoplasmic processes on the surface of activated platelets [12,13]. Electron microscopy illustrated more specific platelet structures [14] such as alpha, dense, and glycogen granules [15,16] (Figure 1). There are several types of platelet granules (alpha (α), dense, lysosomes and peroxisomes) that play particular roles in different platelet functions (Table 1).

Characterization of bioactive components expressed in α-granules (fibrinogen, von Willebrand factor, growth factors and protease inhibitors) demonstrated the presence of different α-granule subtypes [20], suggesting that selective protein packaging takes place during canine platelet ontology, although further studies are needed to understand the specific mechanisms involved in this process [21]. It has been demonstrated that proteins released by α-granules play a pivotal role in numerous platelet physiological activities such as hemostasis, wound healing, antimicrobial host defense, and angiogenesis [22]. In addition, dense granules were characterized showing a content represented by solid electron dense material [23]. The composition of dense granules, comprising ADP and serotonin in major percentage, and other minor components (Ca^++^, Mg^++^ and ATP) suggested a particular role during platelet activation [19].

Platelets also have lysosomes storing proteolytic enzymes (β-hexosaminidase and ß-glucuronidase) that help thrombus remodeling [24].

Canine platelets participate in several events related to primary and secondary hemostasis. Primary hemostasis is a process characterized by platelet aggregation via platelet binding to fibrinogen, leading to platelet plug formation, while secondary hemostasis involves the cleavage of fibrinogen to insoluble fibrin via thrombin generated by the proteolytic coagulation system.

**Figure 2:**
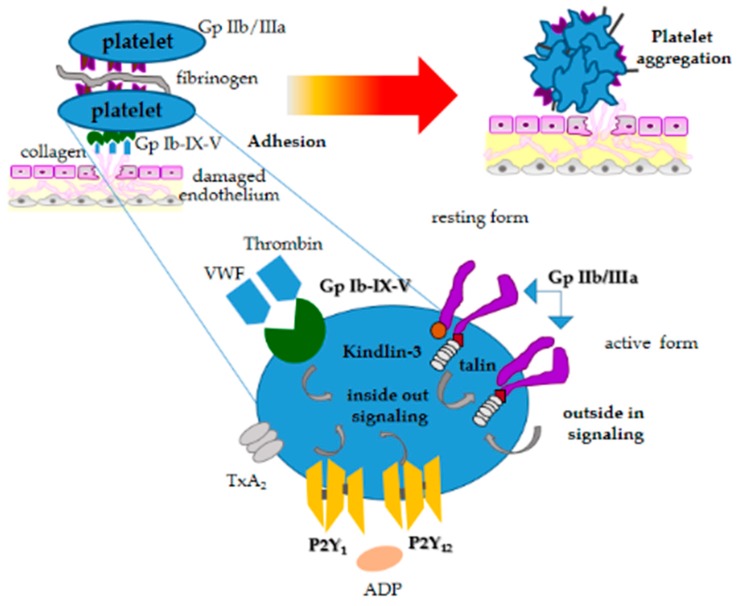
Schematic diagram illustrating the stages of platelet activation.

In primary hemostasis, platelets react in response to vessel wall injury by the exposure of subendothelial collagen. Platelet activation is characterized primarily by platelet shape change, adhesion, aggregation, and granule secretion (Figure 2). These activities are mediated by several cell surface receptors; some receptors are constitutively expressed in active conformation, while others require a particular structural modification or translocation to the platelet surface after initial platelet activation [25]. The first step of platelet activation is shape change, a critical phase during which platelets extend filopodia and generate lamellipodia, causing a dramatic increase in their surface area. Platelet adhesion characterized by adhesion to site of vessel injury follows in close association with shape change. During this process, von Willebrand Factor (VWF) bound to exposed subendothelial collagen primarily binds the platelet surface membrane (glycoprotein receptor Ib-IX-V). Other platelet surface receptors organized as integrins, such as αIIbß3 (GP IIb/IIIa), α5ß1 (fibronectin), αVß3 (vitronectin) and α6ß1 (laminin), can also contribute to platelet adhesion [25].

GPIIb/IIIa is the main receptor involved in platelet aggregation, responding to initial internal signaling generated by platelet agonists binding to their respective membrane receptors.

GPIIb/IIIa binds primarily to fibrinogen and can bind other macromolecular ligands containing the arginine-glycine-aspartic acid sequence (RGD). GPIIb/IIIa in resting conditions has low binding affinity, and its activation requires a signal transduction cascade that leads to the binding of specific proteins talin-1 and kindlin-3 to the β3 cytoplasmic domain, relieving the transmembrane and cytoplasmic domain restraints [26]. Such mechanisms result from signals originating in the platelet cytosol that are transmitted across the plasma membrane (Figure 2).

Other surface platelet receptors such as P2Y12, which is activated by adenosine-5-diphosphate (ADP) (Figure 2), play a particular role in dog platelet activation [27]. Several receptor antagonists have been proven for their therapeutic role [28]. ADP secreted from platelet dense granules plays a particular role to amplify the aggregation signal induced by other platelet agonists, assuring irreversible platelet aggregation. ß-adrenergic receptors have been demonstrated in canine platelets, showing that epinephrine can potentiate aggregation to all agonists in some dogs by involving intracellular calcium [29]. Disruption or deficiencies in any of these pathways can lead to decreased or abolished platelet function, which can be inherited or acquired. These disorders will be discussed later.

Agonist binding and internal signaling then initiates a signaling process, termed “inside-out” signaling, which gives rise to its conformational changes [30] in the GPIIb/IIIa receptor. This conformational modification improves the receptor’s binding affinity for fibrinogen and facilitates interaction with adjacent platelets. Further conformational changes of the receptor create a process termed “outside-in” signaling, that lead to integrin-dependent granule secretion and enhanced aggregation [30].

Platelet granule release is a secretory mechanism where release of soluble factors from activated platelets occurs to facilitate the recruitment of additional platelets to the site of injury and enhance platelet activation and subsequent aggregation. This process is very important for platelet aggregation considering that very often it induces platelets toward an irreversible aggregation.

Dense granules secrete ADP and serotonin, while α-granules release proteins with different functions (adhesive proteins, chemokines, cytokines, coagulation factors, GFs, etc.). Calcium (Cal) and diacylglycerol (DAG)-regulated guanine nucleotide exchange factor I (CalDAG-GEFI) is a signal transduction protein that plays an important role in platelet granule release and integrin activation. CalDAG-GEFI triggers integrin activation through activation of the small GTPase Rap1. The platelet plug generated via primary hemostasis is ultimately stabilized into a firm fibrin clot through the action of thrombin (generated by the coagulation system), which cleaves fibrinogen to fibrin (Figure 2). Activated platelets support the assembly of the coagulation complexes due to the externalization of the plasma membrane phospholipid, phosphatidylserine (PS) and the shedding of small membrane vesicles that express PS. The exposure of PS on the outer platelet membrane provides a negatively charged surface for calcium-mediated binding to negatively charged coagulation proteins.

The development of new investigative analysis systems could provide additional insight into platelet defects not yet fully characterized. The development of proteomic and lipidomic studies [31,32,33] demonstrated the importance of 5974 unique proteins of which only 298 proteins had previous experimental evidence of in vivo expression [32]. Trichler’s study contributed highly in the knowledge of platelet proteome coverage, when compared to the traditional methods [32]. The study of these aspects certainly can offer important information on the composition of platelet proteins and lipids, providing a wide vision of cell and tissue pathways and functions and allow applications also in vascular pathologies (i.e., arterial/deep vein thrombosis and atherosclerosis) [34,35]. More recently, Peng and co-workers (2018) [36] identified lysosphingomyelin (SPC) by lipidomic analysis in mouse and human platelets. This molecule for its attributed role as a novel regulator of platelet degranulation and subsequent thrombus formation offers a new contribution to the knowledge of platelet and demonstrates the feasibility of performing quantitative platelet-lipidome analysis.

These findings expanded knowledge of platelet biology and provided a step towards identifying potential treatment targets and biomarkers for different disease processes.

## 3. Platelet Disorders in Dogs

Canine platelet disorders can affect platelet numbers or function. Platelet disorders can be divided into congenital or acquired thrombocytopenias and congenital or acquired functional disorders (thrombopathias), with acquired thrombocytopenia being the most common in dogs. In addition, causes of thrombocytosis are also described in this section.

### 3.1. Thrombocytopenias

Congenital thrombocytopenias, once considered rare, are now identified with increasing frequency. These platelet disorders are recognized not only based on platelet counts (automated and manual estimation), mean platelet volume (MPV), platelet morphology but also utilizing molecular testing. Routine hematological analysis using automated hematology analyzers frequently detects thrombocytopenia of variable severity in certain breeds of dogs, i.e., Cavalier King Charles Spaniels (CKCS), Cairn and Norfolk Terriers, Greyhounds, and Akita Inu [37,38,39,40,41,42,43].

Macrothrombocytopenia in CKCS is inherited [37,38] and was found in 51% (118.7 × 10^9^/L) [39] and 90% (87.5 × 10^9^/L) [40] of CKCS enrolled in some studies. This platelet anomaly is benign and affected dogs do not exhibit platelet-type bleeding. Two mutations identified in the gene encoding beta1-tubulin, a protein involved in platelet production by megakaryocytes, are responsible for congenital macrothrombocytopenia observed primarily in CKCS [38] and Norfolk and Cairn Terriers [41]. The CKCS mutation has been noted less frequently in other breed lines with least 28 other breeds having this mutation in their lineage; the Cairn/Norfolk mutation has additionally been noted in the Jack Russell Terrier (personal observation). The tubulin mutations induce defective fragmentation of the megakaryocyte cytoplasm, producing decreased numbers of large platelets. Multiple studies conducted on Greyhounds to evaluate breed-specific hematological reference values [42,43] established a platelet count between 80 and 120 × 10^9^/L as normal [43]. The possible causes of these low platelet count in this breed are: (i) the stem cell competition between megakaryocyte and erythroid precursors; (ii) splenic or pulmonary sequestration and; (iii) an inverse relationship with iron stores [44], but an exact cause has not been determined. In another breed as the Polish Hound, an idiopathic, asymptomatic thrombocytopenia with a mild level of thrombocytopenia characterized by an average number of platelets approximately of 167 × 10^9^/L has been described [45]. Recently, Hayakawa et al. (2016) [46] reported a congenital macrothrombocytopenia in Akita dogs based on persistently low platelet counts in the absence of clinical signs and characterized by a unique platelet morphology.

Acquired thrombocytopenias can be present in various disorders and can occur through several mechanisms, including decreased production (infectious agents, drugs, neoplasia and bone marrow disease), premature destruction (that can be immune-mediated and non-immune-mediated), and consumption due to activation/aggregation secondary to disseminated intravascular coagulation (DIC) and non-DIC causes (vasculitis, microangiopathic diseases, turbulent blood flow, etc.). Finally, sequestration (splenomegaly of any cause and pulmonary circulation secondary to sepsis or endotoxaemia) and loss (massive haemorrhage) should also be considered.

Immune-mediated thrombocytopenia (IMT) is the most common cause of severe thrombocytopenia. IMT is a disease in which antibodies bound to the surface of platelets result in their premature destruction by macrophages in the spleen and liver. The principal antibody class associated with IMT is IgG [47,48]. Target antigens for autoantibodies in human beings are epitopes within the glycoprotein (GP) IIb/IIIa or GP Ib/IX complex. GP IIb/IIIa (fibrinogen receptor) has been identified as a target antigen in dogs [48,49,50]. IMT can be classified as primary or secondary based on underlying etiologies. IMT, in the absence of other identifiable disease, is referred to as primary IMT or idiopathic thrombocytopenic purpura [47]. It occurs especially in female dogs [51], and Cocker Spaniels, Poodles, German Shepherd Dogs, and Old English Sheepdogs are over-represented, suggesting a genetic predisposition to IMT in these breeds. Antibodies are thought to be directed against normal host platelet-surface antigens (antiplatelet autoantibodies), and the cause of this antibody production is unknown. On the contrary, in secondary IMT, several initiating factors are detected such as infectious diseases [52], neoplasia, drugs, and blood transfusions. Regarding IMT related to infectious diseases, antibodies that can bind to platelets have been identified in dogs with dirofilariasis [53], angiostrongylosis [54,55] and babesiosis (dogs naturally infected by *Babesia canis* and dogs with experimentally induced *Babesia gibsoni* infection) [49,56,57].

Antiplatelet antibodies have been detected in the serum of dogs with naturally occurring and experimentally induced *Rickettsia rickettsi* infection [58]. In addition, dogs with *Ehrlichia canis* infection can show antiplatelet antibodies in their serum [59,60,61,62]. A secondary immune-mediated thrombocytopenia has also been presumed in dogs naturally infected by *Leishmania infantum* [63,64,65,66] or in dogs naturally co-infected by *Leishmania infantum* and *Ehrlichia canis* [67]. A consequence of these infections is the immune-mediated destruction of platelets caused by exposure of antigenic sites on their surfaces or by immune complex injury to their membranes [68]. Typically, dogs with IMT have normal to increased numbers of megakaryocytes in their bone marrow. Decreased megakaryocytes in IMT may indicate that antibody bound to megakaryocytes contributes to ineffective thrombopoiesis [69].

### 3.2. Platelet Function Disorders

Abnormal platelet function may be primary (inherited) or secondary (acquired). Many of these disorders have been characterized in veterinary medicine (Table 2).

Platelet function disorders should be suspected in animals with an adequate platelet count and evidence of primary (platelet-type) hemostatic bleeding. Patients with decreased platelet function may be clinically indistinguishable from a thrombocytopenic patient.

Primary inherited platelet disorders can be grouped into two categories: extrinsic and intrinsic platelet disorders, often indistinguishable at the clinical level. In extrinsic platelet disorders, platelets are normal, but a protein necessary for their function is either absent, reduced, or dysfunctional [70,71,72]. On the contrary, intrinsic platelet disorders involve the platelet directly and may arise from abnormalities in platelet granules, membrane glycoproteins, signal transduction proteins, or proteins involved in platelet production from magakaryocytes [72]. Buccal mucosa bleeding times are usually prolonged in both types of disorders, while platelet counts and coagulation screening tests are normal, except in inherited/acquired extrinsic platelet disorders (VWD). In these latter disorders, since VWF acts as a carrier for factor VIII, prolongation of activated partial thromboplastin time (APTT) may concurrently occur. In addition, specific tests of platelet function (platelet function analysis using the PFA-100, collagen binding assay) may be abnormal [90].

Petechias, ecchymoses, and mucosal bleeding such as gingival bleeding, epistaxis, and urinary and gastrointestinal hemorrhage can be observed with both types of disorders. Internal bleeding within organs such as kidney, spinal cord, and brain can also occur less commonly. Excessive bleeding during permanent tooth eruption is commonly observed and may be the first indication of the existence of a bleeding diathesis.

The most common type of extrinsic platelet disorder in dogs and people is von Willebrand disease (VWD) [70]. Hereditary VWD is an autosomal trait, characterized by defective or deficient VWF. Three forms of VWD are described: Type I is characterized by an equal decrease in all size of multimers and is the most common type of VWD in people and dogs (many purebreds and mixed breed dogs) [72]. Type II VWD, the second most common type in people, and described for dog species only in German Shorthair Pointers and German Wirehair Pointers [71,73] has qualitative changes in VWF multimers with a selective decrease in large multimers. Type III VWD is the most severe form, characterized by a marked reduction in all multimers; it is rare in people and in dogs has been described as a familial trait in several breeds (Dutch Kooiker dog, Scottish terrier, Shetland sheepdog). There are sporadic cases reported in other breeds (Border collie, Chesapeake Bay retriever, Cocker spaniel, Eskimo dog, Labrador retriever, Maltese, Pitbull) and in a mixed breed dog [72].

Inherited intrinsic platelet disorders have been documented in dogs, including storage pool disorders, abnormalities of platelet membrane receptors, and defects of intracellular signal transduction.

Canine storage pool disorders are cyclic hematopoiesis in Grey Collies and dense granule defects in American Cocker Spaniels [79]. Cyclic hematopoiesis in Gray Collie Dogs is an autosomal recessive disorder, characterized by 12-day cycles of cytopenia. All marrow stem cells are involved, but neutrophils are most affected because of their short half-life [80]. The platelet number does not generally decline below most reference intervals and platelet-dense granules are absent. Clot retraction and platelet adhesiveness are both impaired [91], and excessive bleeding is a potential complication. Platelet dense granule defects have been reported in three families of American Cocker Spaniel dogs, with patients suffering from moderate to severe bleeding after minor trauma, venipuncture, and surgery [23]. This disorder is characterized by normal platelets (in number and morphology) and a decrease in the ADP content and normal content of adenosine triphosphate (ATP), resulting in an inappropriate ratio of ATP to ADP, suggesting a functional dense granule defect [23].

Membrane receptor disorders include Glanzmann thrombasthenia (GT), characterized by a quantitative or qualitative deficiency of GPIIb-IIIa [72]. In patients with GT, platelet aggregation in response to ADP, thrombin, and collagen is absent, despite normal platelet counts and VWF concentration. GT has been best characterized in the Great Pyrenees [81,82], in the Otterhounds (previously called Thrombasthenic Thrombopathia) [83], and more recently in two closely related mixed-breed dogs [84] and in a Golden Retriever [92].

A mutation in the gene encoding the P2Y12 receptor has been identified as a cause of platelet type bleeding in Greater Swiss Mountain dogs [84]. Platelets from affected dogs were not responsive to ADP, but were otherwise normal. Genetic analysis demonstrated a 3 base-pair deletion that resulted in a dysfunctional P2Y12 receptor. Dogs homozygous for this mutation have bleeding tendencies, and the phenotype of carrier dogs is variable [93]. While spontaneous bleeding was not described in the original report, it can be seen with this disorder (personal observations).

Signal transduction disorders have been described in several dog breeds [79,86,87] and suspected in one mixed-breed dog [94]. Inherited signal transduction platelet disorders in Basset hound, Spitz, and Landseer of European Continental Type (ECT) have been well characterized at the molecular level. Distinct mutations in the gene encoding calcium diacylglycerol guanine nucleotide exchange factor I (CalDAG-GEFI) have been identified [72,88]. With these disorders, platelet aggregation responses to ADP and collagen are minimal or absent. Platelet aggregation in response to thrombin is rate-impaired and characterized by a lag phase, lengthening the time to full aggregation to 4–6 min (normal dog platelets typically aggregate fully within 3 min). Because thrombopathic platelets respond to thrombin, clot retraction assays are normal [72].

A kindlin-3 mutation has been discovered in a male German Shepherd dog [89]. The patient’s platelets had a delayed and overall diminished aggregation response to ADP and collagen. Clot retraction was also impaired. Dogs with this mutation have severe bleeding comparable GT, and also show altered leukocyte function and high susceptibility to recurrent infections [72].

Canine Scott syndrome (CSS), an autosomal recessive bleeding disorder, has been described in German shepherd dogs [74,76], and the condition is associated with a mutation in the gene encoding the transmembrane protein (TMEM) 16F (TMEM16F) homolog [75,78,95]. Platelets from CSS dogs demonstrate a lack of procoagulant activity similar to the human disease counterpart, with no evidence of skeletal malformation [75,76,77]. CSS platelets may represent a TMEM16F-null mutant model that demonstrates a central role for TMEM16F in mediating platelet PS externalization in response to activating and death signals [95]. In these patients, platelet function tests (aggregometry, granule release reactions, clot retraction, buccal mucosal bleeding times, thromboelastography), confirm normal platelet function [75,76,77]. The clinical signs observed in this condition are more typical of a coagulopathy due to the inability of the platelets to generate and support a surface for coagulation complex assembly.

The specific pathophysiology of acquired thrombocytopathias is often poorly understood. Underlying disease or drug treatment may result in secondary platelet hyperfunction as well as hypofunction. Abnormal platelet function has been described with various neoplasms, and increased platelet aggregation was detected in two dogs with probable essential thrombocythemia [96]. Hyperglobulinemia, either due to antigenic stimulation (e.g., ehrlichiosis) or neoplasia (e.g., multiple myeloma), is commonly recognized in veterinary medicine as a potential cause of impaired platelet function. The suggested mechanism is the “coating” of platelet surfaces, leading to impaired adhesion and aggregation [97,98]. Acquired von Willebrand disease is a rare bleeding disorder with laboratory findings similar to those of congenital von Willebrand disease. Human cases are associated with lymphoproliferative, myeloproliferative, neoplastic, immunological, cardiovascular, and other miscellaneous disorders. Proposed mechanisms for accelerated removal of VWF from the plasma include specific antibodies to factor VIII/VWF; non-specific anti-bodies forming complexes with VWF, cleared by Fc-receptor bearing cells; absorption of VWF onto malignant cells; increased proteolytic degradation of VWF; and loss of large VWF multimers in high sheer stress conditions. None of the proposed mechanisms are specific for the different underlying disorders [99]. In veterinary medicine, canine hypothyroidism has been shown to exacerbate congenital von Willebrand disease and cause acquired von Willebrand’s disease [100,101]. Canine infection by *Angiostrongylus vasorum* causing acquired von Willebrand disease was reported in a 14-month-old golden retriever bitch [102] and acquired von-Willebrand factor and factor-VIII deficiencies were recently described in an 8-year-old male Australian Shepherd dog [103]. These above case reports may resemble the human condition of acquired von Willebrand syndrome. In addition, enhanced platelet reactivity has also been described in heartworm-infected dogs [104].

In human beings, thrombotic microangiopathies (TMAs) including haemolytic uraemic syndrome (HUS) and thrombotic thrombocytopaenic purpura (TTP) have been also described. TTP is a hypercoagulable state, caused by inhibition of ADAMTS-13, a factor-cleaving protease, by autoantibodies, which leads to a lack of degradation of von Willebrand factor multimers. Von Willebrand factor multimers are prothrombotic, thus ADAMTS13 inhibition leads to microthrombi formation, microangiopathic hemolytic anemia, thrombocytopenia and multiple organ injury, including renal injury. Recently, Maruyama et al. (2014) [105] revealed that the human ADAMTS13 activity ELISA kit is applicable for measurement of canine plasma ADAMTS13 activity and this may improve the knowledge of the pathogenesis of thrombotic diseases also in dogs. In veterinary medicine, a TMA of unknown aetiology, called cutaneous and renal glomerular vasculopathy (CRGV) or “Alabama rot”, has been reported in greyhounds in the USA [106] and in dogs of different breed in the UK [107]. To date, it is currently unknown if CRGV is a novel canine disease or if it is a variant of HUS or indeed one of the other TMAs reported in man.

Several parasitic and infectious diseases have been associated with platelet dysfunction. Reduced platelet aggregation was described in dogs experimentally affected by Rocky Mountain spotted fever [108] and ehrlichiosis [60] and in dogs naturally infected with *Leishmania infantum*, strongly correlated with the clinical phase of the disease [64,109]. Reduced platelet aggregation response to ADP and collagen in dogs co-infected with *Leishmania infantum* and *Ehrlichia canis* has been reported [110]. In subjects treated with anti-leishmania therapy (meglumine antimoniate and allopurinol) and prednisone, a significant improvement in platelet aggregation has also been observed [111], suggesting a pathogenic association between Leishmaniasis and the presence of antibodies against the platelet membrane [112]. Antiplatelet antibodies detected in dogs naturally affected by *Leishmania infantum* [65,66] may cause immune-mediated thrombocytopenia and platelet dysfunction. In addition, previous data reported in people the presence of antibodies against GPIIb-IIIa and other key platelet surface receptors such as GPIb-IX-V [113]. Kristensen et al. [114], in a study in which serum from normal dogs or dogs with IMT was added to platelet-rich plasma (PRP) from a normal dog, found the serum from dogs with IMT causes impaired aggregation to several platelet agonists. In another study, serum from dogs experimentally infected with *Ehrlichia canis* inhibited aggregation of platelets from a normal dog [60].

Acquired thrombopathias have been reported with metabolic disorders. Uremia is recognized as a cause of platelet dysfunction in people, with the underlying mechanism(s) yet to be identified. Neither quantitative nor qualitative abnormalities in VWF have been shown consistently in people with uremia, suggesting that the impaired adhesion in uremic dogs was due to abnormal platelet function [112]. In dogs with renal disease, haemostatic disorders are uncommon, except for some patients being hypercoagulable [115].

Liver disease has been associated with reduced platelet aggregation, although the exact mechanism is unclear. Experimentally induced hyperammonemia has been shown to inhibit platelet aggregation in rats [116,117].

Finally, acquired thrombocytopathias may also be secondary to platelet-inhibiting drugs (aspirin and other non-steroidal anti-inflammatory agents, clopidogrel, certain antibiotics, antihistamines, barbiturates, calcium channel blockers, chondroprotective agents, halothane, heparin, local anesthetics etc.). Acquired thrombopathias are generally reversed when the underlying condition is effectively treated, or drug treatment is discontinued.

### 3.3. Thrombocytosis

Canine thrombocytosis is due to acquired causes and defined by increased platelet production. Platelet production is usually dependent on thrombopoietin; however, it can be thrombopoietin-independent in haemopoietic neoplasia affecting megakaryocytes, e.g., essential thrombocythemia. The most common form of thrombocytosis in sick animals is a reactive thrombocytosis secondary to inflammation, gastrointestinal diseases or neoplasia; the inflammatory cytokines (IL-1, IL-6 and Il-11) observed in these pathological conditions drive thrombopoiesis by stimulating thrombopoietin production [118]. Thrombocytosis can also be due to drugs such as adrenaline that cause splenic contraction. Vincristine increases platelet production and release by the bone morrow. Thrombocytosis can accompany corticosteroid treatment and iron-deficiency anaemias, although the mechanisms for the thrombocytosis in both are unknown [118].

## 4. Platelet Laboratory Testing in Dogs

Platelet-related tests are vital in the analysis of primary hemostatic disorders. Evaluation of a platelet count, combined with a mean platelet volume and blood smear examination, can give useful information with regard to thrombocytopenia. In certain canine breeds characterized by lower platelet counts, the plateletcrit (PCT), which is the volume occupied by platelets in the blood, is an important parameter for hemostatic consideration.

In some clinical situations, PCT may be superior to platelet count as an indicator of primary hemostasis, particularly in dogs with congenital macrothrombocytopenia [119], such as Cavalier King Charles Spaniels (CKCS) [120]. Many CKCS have fewer, but larger, platelets in circulation due to the presence of a beta1-tubulin mutation associated with the breed [121]. The use of PCT is determined by the Advia 120 and 2120, rather than the platelet count to evaluate primary hemostasis in CKCS, reduces the misinterpretation of low platelet mass and risk of bleeding in sick CKCS [122]. Results obtained by Kelley et al. (2014) [120] gave the demonstration that the use of PCT in addition to the platelet count to evaluate primary hemostasis in sick CKCS may help to prevent unnecessary additional testing and treatment by veterinarians. In the same study, Greyhounds’ (a breed with platelets that are relatively low in number and normal in size) platelet count and PCT seemed to function similarly as estimates of platelet mass [120].

Laboratory tests of platelet function can assess platelet characteristics either in whole blood or in platelet-rich plasma (PRP). The proper use of laboratory testing is important for the diagnosis of specific intrinsic platelet function disorders (versus VWD or disorders of secondary hemostasis) and subsequently to provide proper therapeutic recommendations (i.e., treatment with some kind of platelet product versus plasma) in the treatment of various hemostatic disorders [90,123,124] (Table 3).

Platelet function determined by methods, such as light transmission aggregometry performed on platelet-rich plasma (PRP) and impedance whole-blood platelet aggregometry (WBA), represent effective diagnostic tools for evaluating primary hemostasis disorders. These methods measure platelet aggregation response to various agonists [125]. Light transmission aggregometry is generally accepted as the gold standard for evaluating platelet function. Canine platelet response to collagen can vary between people and other animal species platelets as reported in the literature. In fact, a 6-12-fold higher dose of collagen is needed to induce canine platelet aggregation, compared to human platelets [126,127]. Modifications in percentage of aggregation as response sensitivity to different agonists were observed when different common anticoagulants (sodium citrate or acid citrate dextrose-ACD) [128] or snake venom proteins were used as inhibitors of ADP-induced platelet aggregation [128].

Comparison of canine whole blood aggregation [129] with PRP light transmission aggregometry showed differences in terms of response to the employed agonist, suggesting that additional factors such as plasma and blood cells other than platelets affect platelet aggregation and secretion [130].

The development of functional tests such as the new PFA-200 allow identification of antiplatelet drug effect to predict an acceptable decrease in risk for thrombosis [131] (Table 3).

Platelet adhesion assays, based on platelet properties to adhere to specific extracellular matrix components, represent another platelet function testing modality used primarily in platelet function research [132]. In the evaluation of platelet adhesion, an advanced method as the flow chamber has been shown to have some use in dogs [133].

Thromboelastography (TEG) has demonstrated efficacy in evaluating clot formation dynamics, including aspects of platelet function, haemostasis in the dog. Much of the literature regarding TEG application in dogs [134,135,136,137,138] refers mainly to different diseases and to the standardization of the testing method. However, TEG has several limitations (e.g., high HCT false hypocoagulable, low HCT false hypercoagulable, fibrinogen, platelets, etc.) [139]. TEG has also demonstrated a greater utility in identification of subjects showing bleeding signs than typical coagulation profiles [140,141]. In some cases, studies have been performed to evaluate the possible variables influencing TEG tracings [142]. In addition, TEG can be used to evaluate patients with DIC and provide information on individually tailored treatment plans. [143,144,145] (Table 3).

Flow cytometry techniques can determine both organizational and functional aspects of platelets (i.e., platelet size, presence of microaggregates, and surface granularity as well as the P-selectin molecule) [146,147,148]. Recent studies have shown the usefulness of flow cytometry in evaluating platelet activation status by the detection of CD51 and CD41/CD61 [149]. These results showed the ability of flow cytometry to discriminate the influence of different factors (platelet count and platelet agonists) on canine platelet activation [149]. Flow cytometry techniques can aid in the diagnosis of antibody-mediated thrombocytopenia in dogs by detecting platelet-bound IgG on the platelet surface [38,49,63]. This technique has several advantages over other platelet antibody assays in that it allows quantification of antibody binding at the individual platelet level and allows for measurement of low fluorescence levels undetectable by visual observation [150] (Table 3). Recently, new applications of this technique have been introduced to monitor physiological and clinical conditions. In the first case, mitochondria viability related to platelet storing process [151,152,153]. For clinical disease, the technique was demonstrated to be useful to detect directly immunoglobulin associated platelets in both healthy and thrombocytopenic dogs [66,67,154].

Some reports indicate that flow cytometry studies looking at platelet function have limitations due to the complexity of the test procedure, the need for standardization, and quality control [155] (Table 3).

## 5. New Therapies with Canine Platelets 

The introduction of innovative therapeutic approaches based on the use of platelets and their derivatives, such as platelet concentrates, platelet-rich plasma (PRP), and platelet rich fibrin (PRF), for the treatment of disorders of inflammatory conditions such as tendinitis and osteoarthritis, has increased considerably. Growth factors, cytokines, chemokines, and other bioactive compounds present in these platelet products are active in tissue regeneration [156,157]. PRP growth factors include platelet-derived growth factor (PDGF), transforming growth factor-β1 (TGF-β1), transforming growth factor-β2 (TGF-β2), vascular endothelial growth factor (VEGF), basic fibroblastic growth factor (bFGF), and epidermal growth factor (EGF) [156,157,158]. These factors contribute to tissue regeneration by inducing cell recruitment, proliferation, and differentiation processes. The effectiveness of these therapeutic approaches has been evaluated in a variety of diseases by the use of specialized clinic setting that permit sometimes to obtain several advantages, such as the reduction of the costs of effective therapeutic blood derivative, albeit not always with positive results. The use of standardized PRP formulations obtained by commercial kits show some limitations due to a decrease in red blood cells (RBC) and neutrophils [159], which contribute to the clinical platelet product efficacy playing major roles in the inflammatory responses after PRP injection [160]. Carr et al. (2016) [159] suggested to evaluate the amount of growth factors and cytokines in the commercial canine PRP products in order to determine the concentration of platelets and growth factors required for therapeutic effect. Hoareau et al. (2014) [161], compared PRP and buffy coat (BC) protocols for preparation of canine platelet concentrates (PC) and demonstrated that PRP-PC had lesser white blood cells (WBC) and RBC contamination and better platelet functionality than BC-PC. Hlavac et al. (2017) [162] demonstrated the effects of storage on different canine platelet concentrates preparations by comparing the in vitro quality maintenance (metabolic evaluation, cell death, mitochondrial membrane polarization, and activation), with data obtained from humans. The authors, demonstrating similar results, suggested the use of canine platelet concentrates as potential alternatives in veterinary blood banks. Silva et al., in 2013 [18] and after, Parra et al., (2017) [163] confirmed the high efficacy of autologous PC as treatment either for arthritis condition and for avascular necrosis of femoral head in dogs, proposing this therapy as an alternative to the treatments in use for such conditions.

A promising treatment in the regenerative therapy in dogs is mesenchymal stem cells (MSCs), used in combination with platelet products. This innovative approach is based on their specific roles exerted at the injury area in repairing tissue damage. In particular, platelets by secretion of growth factors (GFs) at high concentrations, including transforming growth factor-β, platelet-derived growth factor, insulin-like growth factor, vascular endothelial growth factor and epidermal growth factor, stimulate resident stem cells to proliferate [164]. Simultaneously, the injected MSCs secrete soluble growth factors able to reduce pain and inflammation, increasing blood supply at the site of injury [164]. In canine medicine, this therapy has been evaluated in the treatment of osteoarthritis [165] and tendinopathy [166] with evidence of a synergistic activity between platelets and MSCs due to the capacity of PRP to be a suitable scaffold for cell-based cartilage repair and platelet release of GF, inducing the differentiation of MSCs to chondrocytes (Table 4). Autologous bone marrow (BM)-MSCs’ transplantation onto PRF is also a promising, novel, method for canine osteochondral repair and articular cartilage regeneration [167]. Results derived from the use of canine PRP in various other applications (cartilage, teeth, tendon, skin) demonstrated the ability of platelets to stimulate angiogenesis and participate in new blood vessel development [168] (Table 4).

Important questions remain unanswered regarding platelet product therapies, including the optimal dosing, timing, and frequency of platelet derivate administration for various disease conditions.

Platelet gel is another platelet product, obtained by combining PRP obtained from whole blood with thrombin and calcium or other activators, that has shown to be efficacious in promoting tissue regenerative processes [181]. Questions regarding the ideal biological setting (e.g., percentage of vital bone cells, volume of PRP) for its application require further investigation [182].

The major therapeutic benefit of platelet gel appeared to be mediated by GF released by platelets in promoting essential cellular events involved in wound healing and thus accelerating tissue repair [183].

Two novel approaches in low level laser therapy (LLLT) and prolotherapy have shown to improve the efficacy of PRP treatment for the resolution of pain and lameness [184]. With laser therapy, canine PRP injections are combined with laser therapies (LT) to work in a synergistic manner, improving both cellular regeneration and circulation. Previous studies showed a higher efficacy of PRP and LT used in combination, compared to the techniques used individually, in reducing the healing time of tendon injury in rabbits [185]. This study highlighted multiple roles of laser irradiation in wound healing. Its activity is not limited to a promotion of cell growth by modifications of both mitochondrial physiology and RNA synthesis; it also enhances collagen deposition and reorganization in the affected region by stimulating the release of fibroblast growth factor and increasing the fibroblast cells [185]. Another study has confirmed these findings in dogs [186].

Prolotherapy called “proliferation therapy” or “regenerative injection therapy” (RIT) has proven to diminish pain and resolves clinical signs of lameness [187]. Prolotherapy is based on the injection of sterile nutrient solutions or platelet-rich plasma (PRP) directly into damaged connective tissues (i.e., joints, tendons and ligaments) to promote increased collagen formation. The promotion of stem cells proliferation and differentiation induced by GF facilitate injury repair permit that this therapy may become a viable alternative to surgical options in many cases of Anterior Cruciate Ligament (ACL) and hip dysplasia (HD) [188].

In conclusion, the results of studies evaluating platelet morphology, physiology, biochemistry, and immunology have offered researchers insight into the pathophysiology of platelet-related diseases and new models of therapy.

## 6. Conclusions

Current studies of canine platelets and their function demonstrate potential applications in identifying new therapeutic targets and novel biomarkers and have set a new standard for the resting platelet proteome.

**Figure 3:**
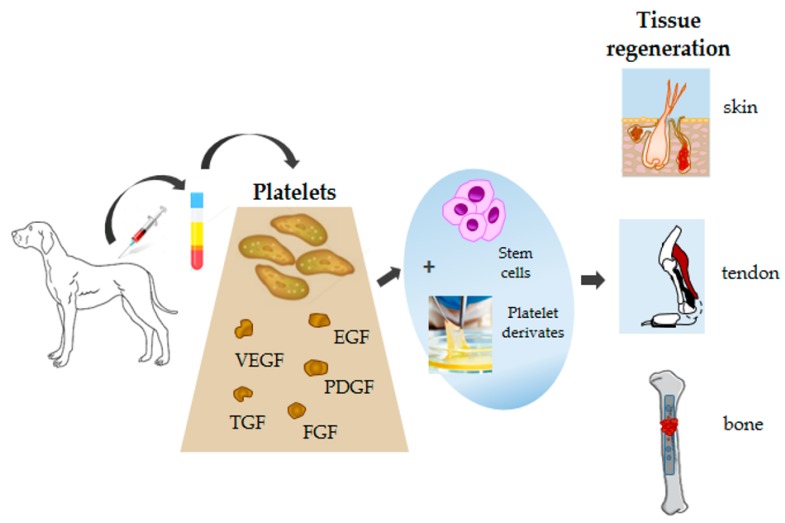
Schematic diagram showing canine platelets, their components, and potential applications in tissue regeneration.

The growing development and use of PRP preparations in wound healing and tissue repair in people will require the development of new randomized controlled studies with large sample sizes to establish therapeutic efficacy (Figure 3). Since dogs are generally considered to be a good animal model for people, human medicine could also potentially benefit from canine research in this area.

## Figures and Tables

**Table 1:** Characteristics of canine platelet granules.

Granule Type	Content	Reference
alpha granules	Fibrinogen Von Willebrand factor growth factors: (1) Insulin-like growth factor-1 (IGF-1) (2) Epidermal growth factor (EGF) (3) Vascular endothelial growth factor (VEGF) (4) Platelet derived growth factor BB (5) Transforming growth factor β1	[17] [17] [17] [17] [18]
dense-granules	Adenosine diphosphate (ADP) Adenosine triphosphate (ATP) Serotonin (5-HT) Ca^2+^ Mg^2+^	[19] [19] [19] [19] [19]

**Table 2:** Canine inherited platelet disorders.

Canine Congenital Platelet Disorders Associated with Bleeding	Alteration Type	Breed	References
**Extrinsic platelet disorders**			
Von Willebrand Disease (VWF)	Defects or deficiencies of VWF (three forms are described) leading to reduced/absent platelet adhesion	Type I: purebreds, mixed breed dogs; Type II: German Shorthaired Pointer and German Wirehaired Pointer; Type III: Dutch Kooiker, Scottish terrier, Shetland sheepdog (familial trait), many sporadic cases in Border collie, Chesapeake Bay retriever, Cocker spaniel, Eskimo dog, Labrador retriever, Maltese, Pitbull and in mixed breed	[70,71,72,73]
**Intrinsic Platelet Disorders**			
Procoagulant expression Scott Syndrome	Lack of phosphatidylserine (PS) expression, membrane microvesciculation failure upon activation with calcium ionophore	German shepherd	[74,75,76,77,78]
Storage pool disorders Cyclic hematopoiesis	Platelet dense granules absence	Grey Collie	[79,80]
Dense Granule Defects	Platelet dense granule defects	American Cocker Spaniel	[23]
Receptors disorders Glanzmann thrombasthenia (GT)	Absence/marked reduction of the glycoprotein receptor IIb-IIIa (GPIIb-IIIa)	Great Pyrenees and Otterhound Mixed-Breed, Golden Retriever	[72,81,82,83,84]
P2Y12	Altered function of the P2Y12 (ADP) receptor on platelet membranes	Greater Swiss Mountain dog	[85]
**Signal Transduction Disorders**			
CalDAG-GEFI platelet disorders	Decreased fibrinogen receptor activation and platelet aggregation to multiple agonists	Basset Hound, Spitz, Landseer dog	[79,86,87,88]
Kindlin-3	Causes decreased/absent activation of beta integrins on leukocytes and platelets	German Shepherd	[89]

**Table 3:** List of methods to test canine platelet function.

Methods of Testing Platelet Function	Sample	Pros	Cons and Limitations
Light transmission platelet aggregometry	PRP	flexible, sensitive to antiplatelet therapy	manual sample processing individual variability
Whole blood aggregometry	WB	easy and time sparing, centrifugation not required, small sample required, maintenance of platelets in their natural milieu	consideration of possible interaction between blood cells
Impedance aggregometry: Multiplate		platelet function under more physiological conditions, good reproducibly to assess platelet aggregation in dogs	Limited hematocrit and platelet count range, Hirudin as anticoagulant to define the optimal concentrations at which various agonists should be used
Aperture closure instruments. Platelet function analyzer (PFA-100, PFA-200)		easy and sensitive to severe platelet defect	rigid closed system, not sensitive to platelet secretion defects and anemia, possible influence by citrate concentration and time from blood collection
Platelet aggregation and ATP secretion	WB	simultaneous response regarding aggregation and ATP content	need to allow the whole blood sample to stand 60 min at room temperature after blood collection
Thromboelastography	WB	higher versatility than traditional coagulation tests	reduced reproducibility, difficult interpretation in subjects with alteration of Hct, platelets, possible request of specialist staff to perform the test
Flow cytometry	WB, PRP, WP	minimal sample required possibility to evaluate resting as well as activated state of the platelets	evaluation of thrombopoiesis, diagnosis of platelet function disorders, and monitoring antiplatelet therapy complexity of the test procedure; need for standardization, and quality control

Platelet rich plasma (PRP); whole blood (WB); washed platelets (WP).

**Table 4:** Applicative use of dog platelets and their derivatives in different tissues or organs for regenerative purposes.

Organ and Tissue Recipients	Platelet Product	Possible Adjuvants Associated	Examined Cases (N)	Reference	Advantages
Bone					
Tibia	PRP	BM − MSCs	10	[169]	Reduction in the time for bone consolidation
		----	65	[170]	Acceleration of bone healing and fracture consolidation
		BM + CHI	30	[171]	Reduction in the time for bone consolidation
Teeth	PRP	Autologous bone	6	[172]	Improvement in bone and cementum formation
Skin	PRP	----	6	[173]	Increase of tissue perfusion and organized collagen bundles
			3	[174]	Increase in angiogenesis, collagen deposition, and epithelization
Tendon	PRP	Adipose tissue derived MSCs	55	[166]	Increase in chondrogenic cells recruitment, cell proliferation, and synthesis of cartilage matrix
Ligament	PRP	----	27	[175]	Reduction of lameness, pain, and effusion
		Leukocyte reduced	12	[176]	Reduction of pain and increase of limb function
		HA	20	[177]	Limb function improvement
Cartilage	PRF	MSCs	12	[167]	Improvement in cartilage regeneration. Increase of proliferation and differentiation of BM-MSCs into chondrocytes
	PRP + Leukocyte	PRF	18	[178]	Improvement in cartilage tissue repair by promoting increased cellular proliferation, extracellular matrix synthesis, and gene expression of chondrocytes
	PRF	----	12	[179]	Improvement in both articular cartilage repair and regeneration
Others	PRP	----	24	[180]	Increase of collagen deposition, improvement in new vessel formation, and overexpression of angiogenic and myofibroblastic genes (COL1α1, COL3α1, VEGF and TGFβ1)

Chitosan gel (CHI); Hyaluronic acid (HA); Platelet-rich plasma (PRP); Platelet-rich fibrin (PRF); Bone marrow mesenchymal stem cells (BM-MSCs).
